# Complete Myocardial Bridging

**DOI:** 10.1016/j.jaccas.2025.104746

**Published:** 2025-08-27

**Authors:** Shaun Abid, Anton Stolear, Samdish Sethi, Ashraf Ahmed, Sachin Goel

**Affiliations:** a Department of Internal Medicine, Yale New Haven Health, Bridgeport Hospital, Bridgeport, Connecticut, USA; b Division of Cardiology, Department of Internal Medicine, Yale New Haven Health, Bridgeport Hospital, Bridgeport, Connecticut, USA; c Division of Interventional and Structural Cardiology, Houston Methodist Hospital, Houston, Texas, USA

**Keywords:** heart transplantation, left ventricular noncompaction, myocardial bridging, ST-elevation myocardial infarction, syncope, ventricular tachycardia

## Abstract

**Background:**

Myocardial bridging is an anatomical variant in which coronary arteries course within the myocardium rather than on the epicardial surface. Complete intramyocardial coronary systems are extremely rare and can lead to significant clinical consequences.

**Case Summary:**

An 18-year-old man with left ventricular noncompaction cardiomyopathy experienced chest pain and syncope while playing basketball, and was subsequently diagnosed with ST-segment elevation myocardial infarction. Coronary imaging revealed a completely intramyocardial coronary system. Despite medical management and placement of implantable cardioverter-defibrillator, he developed heart failure requiring heart transplant evaluation.

**Discussion:**

This case underscores the complexity of managing extensive intramyocardial coronary anatomy, in which conventional revascularization strategies may not be viable and individualized, multidisciplinary approaches are essential.

**Take-Home Messages:**

Extensive myocardial bridging involving the entire coronary system is a rare anatomical anomaly that can precipitate exertional ischemia, arrhythmias, and even sudden cardiac events. Management of complete intramyocardial coronary anatomy requires individualized, multidisciplinary assessment, as traditional revascularization strategies may be unfeasible.

## Graphical Abstract

**Figure:**
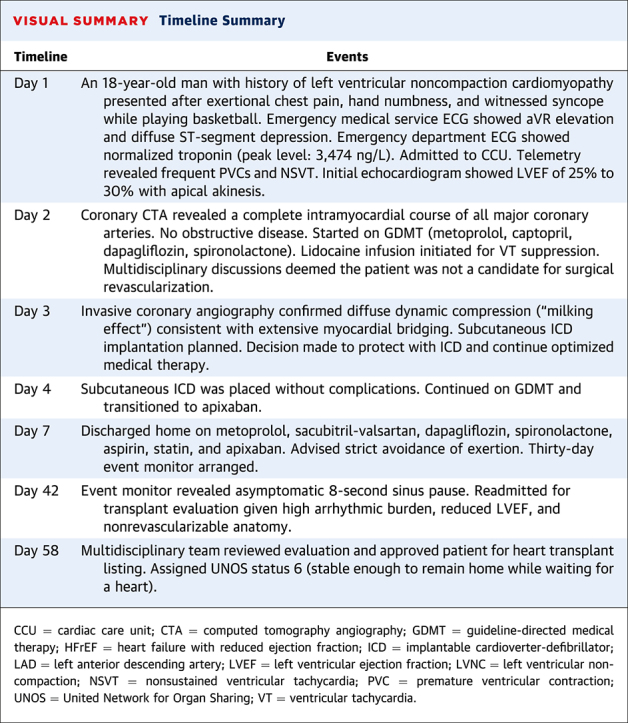

## History of Presentation

An 18-year-old man presented to the emergency department with acute substernal chest pain, bilateral hand numbness, and lightheadedness that began abruptly while playing basketball and culminated in a witnessed syncopal episode. On arrival, he was alert, oriented, and in no acute distress, and his physical examination was unrevealing. He reported no prior similar episodes.

**Take-Home Messages**

• Extensive myocardial bridging involving the entire coronary system is a rare anatomical anomaly that can precipitate exertional ischemia, arrhythmias, and even sudden cardiac events.
• Management of complete intramyocardial coronary anatomy requires individualized, multidisciplinary assessment, as traditional revascularization strategies may be unfeasible.

## Past Medical History

The patient had a history of left ventricular noncompaction cardiomyopathy (LVNC), diagnosed at age 7. Three years before presentation, he was asymptomatic with preserved exercise capacity, and evaluation at that time, including echocardiogram, Holter monitoring, and exercise testing, confirmed LVNC with normal left ventricular (LV) function and no arrhythmias.

## Differential Diagnosis

The differential included LVNC-related arrhythmia or ischemia, coronary anomalies, myocardial bridging, hypertrophic or arrhythmogenic cardiomyopathy, catecholaminergic polymorphic ventricular tachycardia, Brugada syndrome, vasovagal syncope, ST-segment elevation myocardial infarction (STEMI), and pulmonary embolism.

## Investigations

The initial electrocardiogram obtained by the emergency medical service revealed significant ST-segment elevation in lead aVR alongside diffuse ST-segment depressions. Upon arrival to the hospital, the patient's vital signs were notable for a blood pressure of 110/62 mm Hg, a pulse of 102 beats/min, a temperature of 98.3 °F (36.8 °C), a respiratory rate of 12 breaths/min, and an oxygen saturation of 100% on room air. His repeat electrocardiogram was normal. High-sensitivity cardiac troponin T level was markedly elevated at 1,600 ng/L (reference: <12 ng/L) and subsequently peaked at 3,474 ng/L.

Transthoracic echocardiography upon admission revealed severely depressed LV systolic function with a calculated LV ejection fraction (LVEF) of 25% to 30%, global hypokinesis, and akinesis of the LV apex, representing a significant decline from prior studies (Video 1). Continuous telemetry monitoring captured frequent premature ventricular contractions and multiple runs of nonsustained ventricular tachycardia (NSVT) lasting up to 30 seconds.

**Figure 1:**
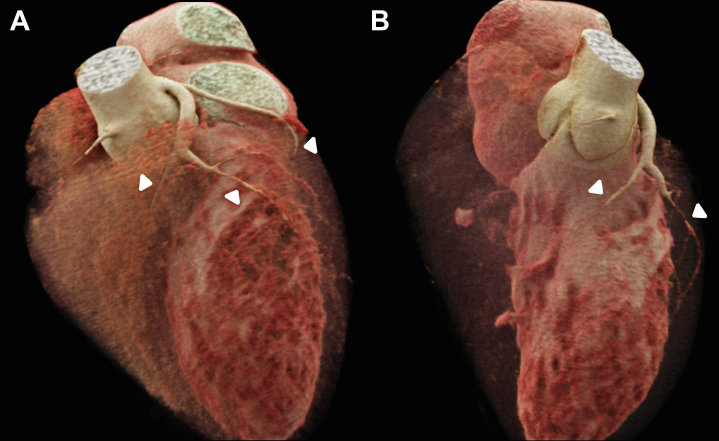
Extensive Myocardial Bridging on 3D Coronary CT Angiography (A) A volume-rendered 3D reconstruction of coronary CT angiography illustrating extensive myocardial bridging. The left anterior descending artery (left arrowhead) gives off a prominent diagonal branch (middle arrowhead) and shortly afterward can be seen tunneling into the myocardium, with nonvisualization of the distal segment. After its origin, the diagonal branch takes an intramyocardial course extending to the lateral and inferolateral wall. The left circumflex (right arrowhead) is significantly smaller in caliber compared with the left anterior descending artery, and it enters the myocardium while continuing to the inferior interventricular groove. No right coronary artery can be seen owing to its deep intramyocardial course. (B) A volume-rendered 3D reconstruction of coronary CT angiography illustrating extensive myocardial bridging from an alternate angle. The left anterior descending artery (left arrowhead) is again seen diving into the myocardium early after its origin, with the diagonal branch following a similarly embedded trajectory. The left circumflex artery (right arrowhead) appears diminutive and courses intramyocardially along the posterior aspect of the heart. As in (A), no visible right coronary artery is appreciated, consistent with a deeply intramyocardial course rendering it radiographically obscured. 3D = three-dimensional; CT = computed tomography.

Coronary computed tomography angiography (CCTA) was performed, revealing a left-dominant coronary system with a unique and extensive anomaly: A completely intramyocardial course of essentially all major epicardial coronary arteries (Figure 1). Subsequent cardiac catheterization confirmed the CCTA findings, showing diffuse dynamic systolic compression of all visualized coronary arteries without obstructive atherosclerotic disease, consistent with the characteristic “milking effect” of myocardial bridging (Video 2).

## Management

Initial management focused on hemodynamic stabilization while addressing the myocardial ischemia and ventricular arrhythmias. Taking the patient immediately to the catheterization laboratory was decided against, as his electrocardiographic changes normalized upon arrival and he was asymptomatic. Guideline-directed medical therapy for heart failure with reduced ejection fraction, which included beta blockade with metoprolol, sacubitril-valsartan, dapagliflozin, and spironolactone, was initiated and titrated. Intravenous furosemide was used for volume management as needed. Given the persistent NSVT, an intravenous lidocaine infusion was required for arrhythmia suppression.

Owing to the severely reduced LVEF, history of syncope potentially related to ventricular arrhythmias, and documented NSVT, the patient was deemed at high risk for sudden cardiac death. A subcutaneous implantable cardioverter-defibrillator was placed for secondary prevention. Multidisciplinary discussions involving the cardiac surgery team concluded that surgical unroofing or revascularization was not technically feasible because of the diffuse and deeply intramyocardial nature of the entire coronary system.

## Outcome and Follow-Up

The patient was discharged on optimized medical therapy and strict activity limitation. A 30-day ambulatory monitor later revealed an asymptomatic 8-second sinus pause, suggesting bradyarrhythmia or sinus node dysfunction from ischemia or medications and prompting readmission. Given his severe biventricular cardiomyopathy, high burden of arrhythmias, anatomically complex features, and few remaining treatment options, the patient was evaluated by the advanced heart failure team. Consequently, he was listed for heart transplantation and assessed as United Network for Organ Sharing status 6, signifying he is stable enough to await an organ at home. He remains on stable therapy while awaiting transplant.

## Discussion

Myocardial bridging, an anatomical variant in which an epicardial coronary artery segment takes an intramyocardial course,^1^ typically involves a discrete portion of the mid left anterior descending artery.^1^^,^^2^ Prevalence of the condition varies significantly by diagnostic modality, ranging from 0.5% to 16% in angiographic series to potentially >30% with CCTA and even higher in autopsy studies.^3^, ^4^, ^5^ Our case, however, represents an exceedingly rare extreme: a completely intramyocardial coronary system identified via CCTA and invasive angiography.^6^ This extensive anatomical deviation presented dramatically with STEMI during intense physical exertion.

The hemodynamic significance of typical myocardial bridging is influenced by factors including the length and depth of the tunneled segment, the presence of associated atherosclerosis, and underlying LV function. These anatomical and physiologic variables can collectively lead to impaired coronary flow reserve, limiting the ability to augment myocardial perfusion during increased demand.^2^ The pathophysiology linking myocardial bridging to myocardial ischemia is multifaceted and primarily involves dynamic coronary compression. While systolic compression is the hallmark “milking effect” seen on angiography, significant coronary flow occurs during diastole. Crucially, studies using intracoronary Doppler and angiography have demonstrated that systolic compression often persists into early diastole owing to delayed relaxation of the overlying muscle.^5^^,^^7^ This diastolic impairment significantly impedes coronary perfusion, especially when diastolic filling time is shortened during tachycardia.^7^ This mechanism is markedly exacerbated by increased sympathetic tone, such as during exercise.^8^ As highlighted in the 2021 *JACC* State-of-the-Art review, heightened sympathetic drive increases heart rate (reducing diastolic time), enhances myocardial contractility (worsening systolic compression and diastolic relaxation delay), and can induce vasoconstriction, collectively blunting the necessary hyperemic response and precipitating a supply-demand mismatch.^5^ This cascade likely triggered our patient's STEMI. Additionally, in bridges involving septal branches, a “branch steal” phenomenon due to pressure gradients across the compressed segment may further compromise regional perfusion.^5^ Given the complete intramyocardial course in our patient, these mechanisms likely acted globally, leading to profound ischemia under stress.

Advanced imaging is crucial for evaluating myocardial bridging. CCTA offers superior anatomical delineation compared with angiography, clearly visualizing the intramyocardial course, length, and depth, and it has a higher detection sensitivity.^4^^,^^5^ Konen et al^4^ reported the prevalence of myocardial bridging to be 30.5% using CCTA, aligning more closely with autopsy findings than the <5% often seen with angiography alone. Whereas CCTA defines the anatomy, invasive angiography demonstrates the functional “milking effect.” Intravascular ultrasound can further characterize morphology and assess for atherosclerosis, which commonly develops proximal to the bridged segment owing to altered hemodynamics, although significant plaque was absent in our patient.^5^ For typical, discrete bridges, physiologic assessment using fractional flow reserve, particularly diastolic fractional flow reserve combined with dobutamine challenge (mimicking exercise stress), is advocated to determine hemodynamic significance, as systolic pressure artifacts can render mean fractional flow reserve unreliable.^5^^,^^8^ However, the utility of focal physiological assessment is questionable in a diffuse, complete intramyocardial system in which ischemia appears anatomically predetermined during exertion.

Standard management for symptomatic myocardial bridging focuses on medical therapy, primarily beta-blockers or nondihydropyridine calcium-channel blockers, to reduce heart rate and contractility, thereby improving diastolic filling and reducing compression.^5^^,^^7^ Nitrates are generally avoided, as they can paradoxically worsen compression and symptoms. This is likely due to intensifying systolic compression of the tunneled artery while vasodilating adjacent nonbridged segments, potentially exacerbating retrograde flow. Revascularization is reserved for medically refractory symptoms. Percutaneous coronary intervention with stenting faces challenges, with high rates of in-stent restenosis, stent fracture, and perforation, especially in long or deep bridges.^5^ Surgical options include myotomy (unroofing the bridged segment) or coronary artery bypass grafting. Myotomy carries risks, particularly ventricular perforation in deep bridges, bleeding, aneurysm formation, and symptom recurrence.^9^ Coronary artery bypass grafting avoids direct manipulation of the bridge but is susceptible to graft failure given the competitive flow from the native vessel, especially when using arterial conduits.^5^^,^^9^

In this patient, the unique finding of a completely intramyocardial coronary system rendered all conventional revascularization strategies (percutaneous coronary intervention, myotomy, coronary artery bypass grafting) technically infeasible or prohibitively high risk. The subsequent clinical trajectory, severe ischemic cardiomyopathy (LVEF: 25%-30%), malignant ventricular arrhythmias (NSVT), and significant conduction system disease (8-second sinus pause) highlighted the devastating potential of this extreme anomaly. This cascade ultimately led to the decision for heart transplantation evaluation and listing, an uncommon endpoint for myocardial bridging but a necessary consideration when conventional treatments are impossible and end-stage heart failure ensues.

The coexistence of LVNC warrants mention. Both LVNC and coronary artery patterning occur during early cardiac embryogenesis. Although many studies have not explored this link, the hypothesis of shared genetic or developmental factors influencing both myocardial compaction and coronary vessel formation is an area for potential future research.

## Conclusions

This report presents an exceedingly rare case of a completely intramyocardial coronary system in an 18-year-old man with LVNC who developed exertional STEMI. Advanced imaging, including CCTA and angiography, was critical in diagnosing this extensive anomaly, which ruled out standard revascularization. The clinical course was marked by severe LV dysfunction, arrhythmias, and conduction disease, ultimately leading to heart transplant listing. This case highlights the importance of considering coronary anomalies such as myocardial bridging in young patients with exertional ischemia, even without traditional risk factors. Although current guidelines provide important frameworks for management, individual patient factors and rare anatomical variants such as found in this case often require a tailored, multidisciplinary approach.^5^ In rare, advanced cases, transplantation may be the only viable option. The hypothetical developmental link between bridging and LVNC deserves further study.

## Funding Support and Author Disclosures

The authors have reported that they have no relationships relevant to the contents of this paper to disclose.

## Appendix

Video 1 Transthoracic Echocardiography Off-Axis Apical 3-Chamber View Demonstrating Wall Motion Abnormalities and Hypertrabeculation The left ventricle shows severely reduced systolic function, with an estimated ejection fraction of 25% to 30% by visual assessment. Regional wall motion abnormalities are present, with akinesis of the entire apex and hypokinesis of all remaining segments. No LV thrombus is visualized. Hypertrabeculation is noted throughout the LV myocardium. Diastolic function is mildly impaired. The right ventricle is not visualized in this view.

Video 2 Right Anterior Oblique Cranial Projection of Coronary Angiography Illustrating the Characteristic Vascular Milking Effect of Myocardial Bridging The video captures the dynamic systolic compression of all visible coronary arteries. The transient extravascular compression during systole forces the abrupt expulsion of dye from the bridged segment, emphasizing its hemodynamic impact.
